# The role of international organizations in equitable and just planned relocation

**DOI:** 10.1007/s13412-021-00698-x

**Published:** 2021-05-13

**Authors:** Gabriela Nagle Alverio, Sara H. Hoagland, Erin Coughlan de Perez, Katharine J. Mach

**Affiliations:** 1 School of Earth, Energy, and Environmental Sciences, Stanford University, Stanford, CA USA; 2 Sanford School of Public Policy, Duke University, Durham, NC USA; 3 Nicholas School of the Environment, Duke University, Durham, NC USA; 4 School of Law, Duke University, Durham, NC USA; 5 Red Cross Red Crescent Climate Centre, The Hague, The Netherlands; 6 Friedman School of Nutrition Science and Policy, Tufts University, Boston, MA USA; 7 Rosenstiel School of Marine and Atmospheric Science, University of Miami, Miami, FL USA; 8 Leonard and Jayne Abess Center for Ecosystem Science and Policy, University of Miami, Coral Gables, FL USA

**Keywords:** Planned relocation, Climate migration, International organizations, Equitable planned relocation, Climate mobility

## Abstract

Since 2010, States party to the United Nations Framework Convention on Climate Change have recognized planned relocation as a viable adaptation to climate change. Planned relocation has been attempted in many communities globally and has raised serious issues of equity in some cases. Implementation driven by principles of equity is crucial in ensuring successful planned relocations that decrease loss and damage. In this Policy Analysis, we put forth a framework for equitable planned relocation rooted in theories of justice as a basis for implementation. The framework centers around three principles: comprehensive recognition of affected stakeholders in decision-making, consideration of socio-cultural risk factors relevant to relocation, and evaluation of multiple measures of well-being. There are many actors involved in planned relocation. Unique features and abilities of international organizations lend themselves to promoting equitable planned relocation in partnership with other stakeholders. Through the exploration of case studies, we identify best practices that international organizations have available to influence the design, implementation, and evaluation of planned relocation processes. These practices are relevant when striving for equity for all affected individuals and communities. Points of intervention include agenda-setting and advocacy, funding and implementation standards, and facilitation of international cooperation. International organizations also face barriers to supporting equitable planned relocation. Limitations include lack of enforcement mechanisms, limited resources, and fundamental dependence on existing governance structures and global collaboration. As the necessity of planned relocations grows, the need for leadership from international organizations in implementation is magnified, underscoring the importance of developing and evaluating approaches to just implementation.

Climate change poses global threats to human security and safety. It is affecting resources such as potable water and fertile land, rendering some territory uninhabitable as a result of sea level rise, and severely impacting infrastructure due to extreme weather events (Kulp and Strauss 2019; McFarland 2017; Scott et al. 2020). As people no longer have sufficient access to critical resources, their mobility will be impacted, with some populations being forced to migrate while others persist in unsafe conditions. Estimates of climate-related migration remain uncertain as associated processes are difficult to model and validate, but current estimates suggest ranges of migrants from hundreds of millions to 1 billion people by the end of the century based on a wide range of climate impacts (McLeman and Hunter 2010; Hsiang and Sobel 2016; Rigaud et al. 2018). The question that remains unanswered is not *if* climate-induced mobility will be a reality, but *how* it will occur, thereby highlighting the important role of proactive planning by responsible actors.

Since 2010, States party to the United Nations Framework Convention on Climate Change (UNFCCC) have recognized mobility via planned relocation as a viable adaptation to climate change. Planned relocation is a response to environmental and climate hazards in which people move, on their own or with assistance, to a new location with the ability to rebuild their lives (UNHCR 2016). Although we focus on environmentally driven relocations, it is important to note that relocations can be driven by a host of issues, including development, conflict, and politics. We will discuss a few examples, especially related to development, where relevant for climate and environmental relocations. Managed retreat is a subset of planned relocation that focuses on mitigating the impacts of sea level rise and other hazards and then returning the land to open space (Neal et al. 2017).

Planned relocation and its subsets are specifically relevant to decreasing the human impacts of climate change and its associated loss and damage (Barnett et al. 2016). Governments, non-governmental organizations (NGOs), and international organizations have implemented planned relocations to date in an effort to reduce the suffering brought about by climatic and environmental impacts. In a number of cases, equity issues have been raised in both the process and the outcomes of the planned relocation (Table 1). In some cases, the affected communities were not involved in the decision-making or were forced to relocate, leading to dissatisfaction, poor health outcomes, and decreased livelihoods (Vithanagama et al. 2015; Dannenberg et al. 2019; Piggott-McKellar et al. 2019; Robinson 2003). Even when some community members were consulted, in some cases, women were left out of the conversation and experienced worse outcomes as a result (Arnall et al. 2013; Piggott-McKellar et al. 2019). In others, government inaction or mistakes have caused critical relocations to be put on hold indefinitely or to lead to inequitable outcomes for residents of the same background in terms of their livelihood prospects or access to critical resources (Bronen 2011; Dannenberg et al. 2019; Mortreux et al. 2018; Albert et al. 2017; Niven and Bardsley 2013).

**Table 1:** Examples included here are coordinated relocations involving significant amounts of planning. These examples include planned relocation in response to climate change and the environment as well as development-driven resettlements, where relevant for understanding equity. The cases were selected based on a search for planned relocation in the context of climate change or the environment and were included if evidence of issues of equity was found in existing documentation. Cases were selected to represent a variety of geographical contexts as well as implementing actors. Relevant categories from our framework for equitable planned relocation are in the “Issue” column as follows: *RS*, recognition of stakeholders; *SR*, incorporation of socio-cultural risk factors; *WE*, well-being-based evaluations. Acronyms used: multilateral development banks (MDBs), international non-governmental organizations (INGOs), international organizations (IOs), non-governmental organizations (NGOs), International Federation of Red Cross and Red Crescent Societies (IFRC), and United States Agency for International Development (USAID)

Location of the planned relocation	Approx. dates	Project details	Actors involved in implementation	Issue relevant to equity
West Bengal, India (Mortreux et al. 2018)	1977	Government withdrew funding for services on the Ghoramara and Lohachara Islands due to erosion. New regime introduced a resettlement plan.	West Bengal State Government	People were relocated to areas with less land and no access to fishing (their primary source of income), and the new land was not able to be cultivated in the first few years. There were also equity issues in the distribution of relocation entitlements over time. *SR*, *WE*
Narmada River, India (Robinson 2003)	1987–2002	Sardar Sarovar dam and irrigation complex built, displacing 320,000 people.	India federal government, World Bank (later withdrew support)	Local communities were not involved and protested. Government exaggerated the benefits of the project in comparison to the number of displacements and lost livelihoods. *RS*, *SR*
China (Robinson 2003)	1994–2009	A total of 1.2 million people displaced due to the Three Gorges Dam project.	China federal government, World Bank (later withdrew support)	No participation of the affected people in the decision-making and inadequate compensation given. *RS*
Laos (Baird and Shoemaker 2007)	1997–2007	Forced transition from upland agriculture to lowland paddy rice cultivation.	Lao Federal Government, IOs, MDBs, bilateral aid agencies, and INGOs	Promises made by the government to encourage move were not kept. Voluntary resettlements were coerced. In the new land, villagers were no longer able to grow enough food to survive, and there were few alternatives. Poor health outcomes also associated with the move. *RS*, *WE*
Sri Lanka (Vithanagama et al. 2015)	2004	Eighteen families relocated following a tsunami.	Sri Lankan Federal Government, IFRC, USAID, local NGOs	No community involvement in decision-making leading to sentiment among residents that outcomes were unjust. *RS*
Cagayan de Oro, Philippines (Franta et al. 2016)	2011–ongoing	Aftermath of tropical storm Sedong led the government to implement planned relocation for 14,000 families.	Local government	Tens of thousands of people are still waiting to be relocated. Many of those who have been relocated struggle to make a living. Some sites are in hazardous areas or lack basic services like water. *WE*
Lesotho (Robinson 2003)	1993–1998	Dam construction displacing over 27,000 people.	Lesotho federal government, World Bank, financial institutions	Disproportionate negative impacts on Indigenous people and low-income groups. World Bank said, “the results on the social side… are clearly distressing” (Robinson 2003). *SR*
Chicomo Locality, Mozambique (Arnall et al. 2013)	2000	1200 adults relocated following floods.	Local government, local NGOs, national NGOs	Said to be voluntary, but the government denied livelihood support if people did not move. After the resettlement, most residents reported dissatisfaction due to difficulty earning a living, and many moved back to the flood-prone areas. Women reported higher dissatisfaction rates than men. *RS, SR, WE*
Byron Bay, Australia (Niven and Bardsley 2013)	1988	With community consultation, a relocation plan was implemented to have housing removed as it is affected by erosion.	Byron Bay local government	Enforcement of planned retreat has not been consistent across community members. *RS*
Nuatambu, Solomon Islands (Albert et al. 2017)	2007–2016	In total, 133 people have relocated to 12 separate sites due to sea level rise with no involvement from outside actors.	No other actors	Fracturing of community. Disproportionate negative impacts on the elderly with regards to sanitation, access to drinking water, and transportation; *SR, WE*
Denimanu Village, Fiji (Piggott-McKellar et al. 2019)	2012–2014	Nineteen households hit by a cyclone relocated.	Fiji Federal Government	No recognition of community members in the decision-making process. Women felt especially marginalized from the process due to compounding societal and cultural norms. Communal infrastructure was not built, and there were poor health outcomes for women. *RS*, *SR*, *WE*
Cartaret Islands, Papua New Guinea (Dannenberg et al. 2019)	2019–present	Relocation of 2700 people facing coastal erosion and sea level rise.	Local NGO, Catholic church	Challenges to livelihoods in the new site and disputes over fishing access; *SR, WE*
Kivalina Village, Alaska (Bronen 2011)	1998–present	Villagers voted to relocate due to erosion causing loss of infrastructure.	United States Federal Government, Alaska State Government	Until 2018, the government would not approve the proposed relocation sites. In 2018, an access road and school was funded, but the government remains unaccountable for further relocation efforts. *RS*
Gardi Sugdub Island, Panama (Dannenberg et al. 2019)	2015	A total of 1,000 Indigenous people relocated due to sea level rise.	Local government, Panama Federal Government	Linked to negative health impacts due to increased malaria risk. Despite government promises, progress has been slow, causing some people to move away. *WE*
Isle de Jean Charles, Louisiana (Dannenberg et al. 2019; Georgetown Climate Center 2020)	2016–present	A total of 80 Indigenous people of two tribes have organized their relocation in partnership with the government due to sea level rise and erosion.	US Federal Government, Louisiana State Government	Poor health outcomes because slow processes have led to some people moving elsewhere, and residents feel the relocation does not meet the tribes’ unique needs. *RS, SR*

The recurrence of equity issues in planned relocation has resulted in power structures being reinforced that lead to the unequal treatment of marginalized groups (Mortreux et al.^2018^). Secondly, these outcomes lead to a trust deficit between people and governments, thereby increasing the time and resources needed on the part of governments to implement planned relocation as a proactive adaptation (Ajibade 2019). In response to these equity issues, there is a need for a framework that highlights priorities for equitable planned relocation that actors in the space can utilize when implementing planned relocation.

In Table 1, we have identified examples of historical planned relocation efforts with issues of equity. The cases were selected based on a search for planned relocation in the context of climate change or the environment and were included if evidence of inequity was found. We categorize each based on the relevant category for improvement of equity based on our framework for equitable planned relocation. The categories include recognition of stakeholders (RS), incorporation of socio-cultural risk factors (SR), and well-being-based evaluations (WE).

## A framework for equitable planned relocation

Justice has been approached through multiple disciplinary, epistemological, and theoretical vantage points. In synthesizing this framework, we deliberately draw from a variety of viewpoints to combine those most relevant to planned relocation. We have identified the following three priorities as most important due to their ability to counteract context-specific power imbalances and existing inequities. Additionally, we crafted this framework to address inequality before, during, and after implementation. Importantly, in applying a lens of equity, this framework is most relevant to voluntary planned relocation efforts. However, in cases involving complicated factors beyond the control of actors affected by relocation, this framework remains relevant in the implementation process.

### Recognition of affected stakeholders

Development-driven relocation literature has brought forward the importance of participatory processes grounded in the agency of affected communities (Draper and McKinnon 2018). Just climate adaptation aligns with this literature as it requires the involvement of stakeholders in decision-making, especially when they are socially vulnerable (Malloy and Ashcraft 2020). Thus, recognition, defined as the inclusion of often marginalized groups that are typically left out of decision-making, is a key component in implementing equitable planned relocation (Schlosberg 2012; Young 1990; Fraser 2014). Without recognition, decisions related to planned relocation can be made based primarily on economic profitability and lead to the social and political isolation of low-income or otherwise marginalized groups (Nygren and Wayessa 2018; Table 1). Importantly, attempts at recognition can stray into tokenism in which involvement is asked for but not acted upon (Ocloo and Matthews 2016). As such, success in this realm goes beyond informing or merely consulting affected parties. It consists of documented decision-making that is changed based on the input of stakeholders, whether in a partnership or through the delegation of power (Guaraldo Choguill 1996). A holistic, participatory process involves considerable amounts of time and resources in order to take cultural and linguistic factors into the engagement process (Bronen 2011). This highlights the need for proactive responses to climate impacts by responsible actors.

Importantly, even with ample time and resources, the process of recognition can result in cases in which varying priorities arise. Communities are not a monolith. The opinions that are voiced, in some cases, may be difficult to incorporate and, in others, may be irreconcilable in a single solution (Zakus and Lysack 1998; Table I). This may mean that rather than developing a one-option plan for relocation, organizations utilize the feedback they receive to develop a flexible plan that includes multiple options for the planned relocation of a community allowing individual households to determine their best option. For example, a plan with the options for resettlements both near an urban center, which provides a variety of livelihood prospects, as well as in an area with available farmland, allows for families with different skillsets to choose the option that suits them best. Actors should engage in existing community processes and governance structures in developing these multiple options that each meet the threshold for risk mitigation that governs the project and balance the tradeoffs that community members face.

### Incorporation of socio-cultural risk factors

Just adaptation requires the explicit recognition of causes of systemic injustice (Malloy and Ashcraft 2020). Therefore, it is critical that socio-cultural risk factors, defined as social vulnerabilities rooted in culturally defined power structures, are incorporated into the planning process (Dannenberg et al. 2019). This incorporation is a necessary precondition for enabling distributive justice. In the context of climate mobility, this entails the equitable sharing of burdens and benefits that arise from the actions taken by relevant actors (Kuehn 2000). In practice, what this typically means is not that every individual receives an *equal* share of the outcomes, but that those who are affected disproportionately by the environmental risk due to their socio-cultural risk factors, such as being elderly, being a person of color, being a girl or woman, or having livelihoods that are invested in the at-risk location, receive an outcome that takes these factors into account (Kuehn 2000; Herrmann and Sauerborn 2018; Arnall et al. 2013). For example, an outcome that factors in both the accessibility to education for young girls and the livelihood prospects for women based on their skillset is one that incorporates several socio-cultural risk factors. Actors implementing planned relocation should take into account both those who will benefit from adaptation efforts and those who may be harmed by them, with a lens for the long-term outcomes as well as the short-term risk reduction.

This incorporation is an ongoing process that should begin with the assessment of the socio-cultural risk factors that divide the community, continue in the recognition process to ensure that marginalized members of the community have their voices heard, and remain a part of each decision made thereafter. Importantly, there is a need for further research and investment into specific processes that promote recognition via the incorporation of socio-cultural risk factors in a context as complex as planned relocation. It will undoubtedly be context-specific, but will need to involve sub-community relationship building, assessments, and meetings that run parallel to the larger community proceedings. It is almost certain that the needs of those with more socio-cultural risk factors will be different from the needs of others. Consider the needs of an elderly Indigenous woman as opposed to that of a young dominant-culture man. The differences in their experiences and needs further highlight the importance of a solution with multiple options that specifically address the losses incurred as a result of relocating for those sub-communities. The need for a conflict resolution strategy becomes even more critical when incorporating socio-cultural risk factors because often consensus will not arise. A balancing of perspectives, across identities and timescales, through a deliberative context-responsive process is therefore essential to ensuring that dominant power structures do not further marginalize sub-community members (McWhirter et al. 2014; Bowmer 2007; Zhang and Fung 2013). We do not suggest that planned relocation efforts must right all historical and structural wrongs. However, without actively taking these conditions into account, they will likely be reinforced or exacerbated in the process rather than challenged and addressed Ajibade (2019).

### Evaluation based on multiple measures of well-being

The evaluation of planned relocation efforts is critical to informing future actions in the affected communities as well as in other projects entirely. An equitable evaluation takes into account multiple measures of human well-being, along with the economic and environmental outcomes of the relocation (Bronen 2011). This means that actors must identify, incorporate, and evaluate success upon measures of well-being that best capture the human outcomes of the process in both the moving and receiving communities. The measures should aim to ensure that the new living conditions are comparable to or better than where people began (Georgetown Climate Center 2020). This starts with respecting previously recognized rights to things like housing, safe drinking water, and clean air (Bronen 2011; Harris and Symons 2010). Following these basic requirements, factors like access to socioeconomic opportunities (e.g., education and formal employment), public health infrastructure (e.g., nutritional food and hospitals), and governmental responsibilities (e.g., public utilities and political efficacy) should be incorporated into the evaluation (OPHI 2015; UN 2015). Additionally, vulnerability to future disaster or conflict and community cohesion should be measured longitudinally as well as in the short-term (IOM 2017). Actors in the space should compare the new living standards across the aforementioned factors to the old ones and to agreed-upon basic standards of human living conditions, especially in the case that the old site does not meet basic standards, in the evaluation process.

In a process as challenging as planned relocation, there is almost certainly going to be loss on some levels of well-being. The process of planning should be directed by the affected people and involves a weighing of tradeoffs related to the aforementioned factors (Tuhkanen et al. 2018; Hardoy et al. 2011). Tuhkanen et al. (2018) have proposed a typology for tradeoffs in implementing planned relocation that takes into account aggregation, risk, equity, time, and participation. After the move, there will still likely be different outcomes among community members, providing opportunities for follow-up action targeted to specific factors of well-being (Leape 2020). Through this analysis and follow-up, actors take active steps towards ensuring that the affected parties build back better in the ways that are most important to them and reduce multiple dimensions of risk after the relocation.

## Positioning international organizations

Among the many actors involved in planned relocation, international organizations are uniquely positioned to promote equitable planned relocation in partnership with other stakeholders. We focus here on examples that are most relevant to organizations with multiple member states, including bilateral and multilateral organizations. Although not explicitly highlighted, international NGOs (INGOs) and national-level agencies that work in the planned relocation space are also germane.

International organizations have exhibited commitment, leadership, and action around climate adaptation and risk reduction (World Bank Group 2019; UN 2017; OECD 2019). Specifically, planned relocation has been a growing part of the climate adaptation and development landscape for international organizations. The Sendai Framework for Disaster Risk Reduction adopted by UN member states in 2015 directly mentions planned relocation as a solution. The World Bank has funded and partnered with government efforts to implement development-driven resettlement in many countries, including Mozambique, South Africa, and Uganda (Table 1). International organizations play a unique role in this regard because they are positioned both to act proactively to approach risk management and to address complex issues such as the loss of communal sovereignty that can occur amid planned relocation.

Along with their commitments to action on climate adaptation, international organizations have a history of action and success in promoting equity. Many international organizations have provided principles, policies, and frameworks that serve as examples for the global community. For instance, the UN Convention on the Elimination of All Forms of Discrimination Against Women adopted (CEDAW) in 1979 established principles of equality and has led to the striking down of many laws that upheld the discrimination of women (Global Justice Center 2017). CEDAW joins a long history of UN bodies and treaties that have upheld equity by way of protecting human rights (e.g., ICERD, ICCPR, ICESCR, CAT, CRC, ICMW) (OHCHR 2020). Along with policies, international organizations have funded and implemented many equity-promoting programs such as the World Bank’s District Upland Development and Conservation Project in Laos and the United Nations Children’s Fund (UNICEF) Social Protection Program (World Bank 1999; World Bank 2004; Wang and He 2019).

Specific to the intersection of equity and planned relocation, international organizations have committed to preventing involuntary resettlement. For instance, the Interamerican Development Bank (IDB) has a policy that provides special consideration regarding the risks of impoverishment associated with relocation when working with low-income groups (IDB 1999). The World Bank, Organization for Economic Cooperation and Development (OECD), and several branches of the UN have similar policies around preventing inequity in resettlements (World Bank Group 2019; OECD 1992; UNHCR 2011). In doing so, these organizations have established a set of best practices regarding equity that they hold their partners accountable to.

As such, international organizations and their partners have made great strides towards equitable adaptation thus far. Nevertheless, the problems that the global community faces in this realm are enduring and require sustained action. Importantly, the massive moment of disruption the globe is facing as a result of the COVID-19 pandemic has the potential to threaten this progress and further undermine equity. It is therefore critical that international organizations leverage their unique features and abilities to promote equitable planned relocation.

## Points of intervention by international organizations

Out of many features and abilities held by international organizations, we highlight three that are most relevant for promoting planned equitable relocation. Table 2 provides a representation of the points of intervention by international organizations as mapped onto the framework for equitable planned relocation and examples of implementation for each.

**Table 2:** Unique features of international organizations, as relevant to equitable planned relocation and examples of each. The horizontal categories refer to the framework for equitable planned relocation and the vertical categories refer to the points of intervention by international organizations

	Recognition of affected stakeholders	Incorporation of socio-cultural risk factors	Well-being-based evaluations
Agenda-setting and funding	IOs can incorporate recognition into the priorities of planned relocation, and some can make funding contingent upon it. E.g., create a toolkit for engaging affected stakeholders in decisions on planned relocation, and make funding contingent on the implementation of the toolkit and demonstration of the decisions made by stakeholders.	IOs can set an agenda that specifically focuses planned relocation on communities that would like to relocate and are most impacted by other systems of oppression and vulnerabilities. E.g., create multiple relocation options that can be selected by people with different risk factors, e.g., age, race, family structure, and gender. This should include different packages of physical infrastructure and soft skill development that each meet the standard of sufficient risk mitigation.	IOs can include well-being-based evaluations as part of the priorities of planned relocation, and some can make funding contingent upon it. E.g., incorporate ongoing and longer-term evaluation measures to determine the outcomes across well-being over time. Design contracts to include prioritization of and funding for results of evaluations to be acted upon in future relocations.
Best practices and accountability	IOs can promote recognition as a best practice and hold actors accountable to including affected stakeholders in the decision-making process. E.g., operationalize community participation frameworks, integrate deliberate processes for conflict resolution, and incorporate feedback on the process from community members into the evaluation of the project (Burns et al. 2004; Millner et al.^2019^; Paul 1987).	IOs can promote the incorporation of socio-cultural risk factors as a best practice and hold actors accountable to it. E.g., develop and disseminate a holistic assessment framework of socio-cultural risk factors that takes into account quantitative and qualitative factors driven by the affected community members, e.g., gender, income, ethnic-groups, and religion.	IOs can develop a framework for well-being-based evaluations through the recognition process, promote it as a best practice, and hold actors accountable to utilizing it. E.g., include strategies for the sharing and transfer of knowledge (e.g., EIM) through all stages of the planned relocation project and across organizations (Boh 2007; Almeida and Soares 2014). Hold organizations accountable for responding to the knowledge gained in future projects through institutionalized reporting mechanisms.
Facilitate coordination	IOs can include affected stakeholders in the process through their facilitation of coordination across borders or organizations. E.g. in the case of challenges regarding sovereignty, e.g., Indigenous peoples or island nations, provide support in negotiations for territory-less nations to maintain decision-making power and governance structure to the extent that is possible.	IOs can facilitate the coordination between organizations that are well-equipped to incorporate socio-cultural risk factors, like local NGOs, and governments. E.g., make connections and build relationships with local organizations based in the affected communities, especially those focused on issues of social justice that are not directly linked to climate, e.g., gender equity, racial justice, and youth justice.	IOs can facilitate the coordination between organizations in order to create well-being-based evaluations that may differ from their normal program evaluations. E.g., host a workshop with governments and international and local NGOs focused on the development of well-being-based evaluations with specific and generalizable concepts for utilization by actors on short-term and long-term outcomes of planned relocations.

### Agenda-setting and funding

International organizations play a key role in setting the international agenda, which can guide the attention of governments, NGOs, and other international organizations towards coordinated action. They can identify gaps and advocate for an equitable approach to planned relocation. The reach of international organizations in agenda-setting extends from applications such as implementing planned relocation on the ground, redefining sovereignty for nations who have lost their territory, and abiding by principles of justice through the centering of Indigenous peoples.

International organizations have had many successes in agenda-setting. A key example is that of gender mainstreaming, which came out of the Beijing Platform for Action at the 1995 UN Conference on Women and led to the prioritization of gender equality within the UN, its member states, and the broader NGO and philanthropy field (Moser and Moser 2010). A testament to gender mainstreaming’s utility as an international agenda item is the success of the European Union in incorporating it across many levels of government by way of structural funds, development, employment, research, and education (Pollack and Hafner-Burton 2000).

Some international organizations can push forward the agenda they set through the provision of funding. In 2019 alone, the World Bank committed $50 million to an Action Plan on Climate Adaptation and Resilience. This funding will allow the World Bank and its partners to meet climate adaptation goals far sooner and in a more coordinated manner than without funding. In addition to the direct funding of projects, international organizations can provide funding for projects contingent upon the implementation of policies that promote equity around climate mobility (Harris and Symons 2010). Such funding would better support countries in addressing the flows of internal migration that are predicted to far exceed external migration. To achieve this end, international organizations can partner with one another and host governments in creating funding platforms for greater scope and scale (Georgetown Climate Center 2020). Importantly, the commitment by international organizations to defund projects when they stray from the principles of equity in the space of planned relocation is well-established (Table 1).

International financing is critical to supporting governments in affected low-income countries in implementing equitable planned relocations. They are at double exposure for experiencing financial pressures from both globalization and climate change, increasing the necessity for planned relocation (Warner et al. 2013; Thorpe and Figge 2018). As such, the choice by international organizations to provide funding to countries that would otherwise not be able to implement planned relocation efforts promotes local and international equity. This is exemplified by the ongoing West Africa Coastal Areas Resilience Investment Project funded by the World Bank, the International Development Association, and the Nordic Development Fund that provides $210 million to coastal erosion projects whose impacts will reach over one-third of the West African population and 42% of GDP generating activities (Seck 2018).

### Disseminating best practices and establishing accountability

Each time a government or other local actor implements a project, there are lessons to be learned that would benefit governments around the world in future projects. Given where they sit in the global structure, international organizations are in a position to amass the best practices as learned through previous trials of planned relocation and share them with networks of practitioners, governments, and other interested stakeholders. More importantly, given the specific context of each planned relocation, there are limits to the applicability of detailed best practices from singular case studies. International organizations must therefore referee the dissemination of specific lessons through the lens of the context by which it was gained. There is a long history of inequitable planned relocations, some of which involved international organizations and others which did not, whose lessons were likely not readily available to governments who later had similar equity issues in their implementation (Table 1). It is therefore critical that a shared understanding of the possible roles of each stakeholder and implications for the whole system be created (McNamara et al. 2018). Although international organizations are well-positioned to facilitate this scheme of best practices, it will take active coordination and planning on their part to promote triple-loop learning, which works to unlearn the underlying assumptions around issues of inequity, as required in these cases (Gupta 2016).

Along with the examples listed in Table 1, a plethora of planned relocation efforts not linked to climate exist in which equity issues have predominated. Some may have been due to outright negligence. Others may have had the intent to relocate marginalized or stateless groups such as Indigenous peoples or religious minorities for political purposes. Even still, many have likely been carried out by well-meaning actors who faced the challenges of balancing organizational priorities, community needs, funding constraints, and a lack of a historical standard for transparency and accountability. As a necessary counterpart to disseminating best practices, international organizations are well-positioned to fill this gap and promote transparency, encourage best practices, and create accountability in order to establish a baseline for equitable action in the space.

This is critical because the historical baggage that connotes planned relocation and development-driven resettlements is not one that will be easily undone. Indigenous peoples in the USA have a history of enduring brutal forced relocations that aimed to destroy their cultures. The associated traumas are passed down intergenerationally and significantly impact the physical and mental health of all generations (Walls and Whitbeck 2012). With histories of planned relocation tainted with gross injustices, the role of international organizations in holding themselves and other actors accountable to promoting equity is critical to preventing the repetition of history.

### Facilitate coordination among relevant actors

One of the most powerful features of international organizations is their ability to facilitate coordination among a wide variety of actors. For instance, international organizations often operate refugee camps and must coordinate with local and national governments, international organizations and NGOs, and local groups and NGOs. In Bangladesh, the Rohingya refugee camps were coordinated by the United Nations High Commissioner for Refugees (UNHCR). It was not only facilitating the day-to-day activities but also working with the government of Bangladesh to strategically plan for the future of the camps and those in them (Milton et al. 2017). In coordinating with local actors, international organizations also remove themselves from the position of acting without a full grasp of community dynamics that will be incredibly difficult, if not impossible, for them to understand.

This feature is especially relevant for international migrants who face a host of political and social concerns as a result of their migration, including but not limited to endangered livelihoods and poor physical and mental health outcomes; diminished political efficacy; and reduced access to critical resources (Tschakert and Tutu 2010; McLeman 2017; Schwerdtle et al. 2018). Migrants also risk being labeled a national security concern by increasingly anti-immigrant regimes, leading to an exacerbation of the negative impacts. Given experiences with additional border restrictions during the COVID-19 pandemic, international organizations have the opportunity to coordinate relocations within and across borders that specifically mitigate the aforementioned impacts.

International organizations also hold the unique power of negotiating international agreements, which can include equitable climate mobility solutions. A seminal exemplar is the Inter-state Consultation Mechanism on migration in the Pacific region (PIDC) facilitated by the International Organization for Migration (IOM). The PIDC includes 21 states and territories that seek to develop policy around migration flows. Together, they have sought to address immigration processes within their borders in the wake of a natural disaster by specifying the role of immigration officers, delineating the separation of individual documentation and disaster relief, and prioritizing proactive planning (PIDC 2010). The PIDC includes member states and territories that might otherwise be left out of the conversation on migration policy in the region yet are largely affected by it. In coordinating the convening of the PIDC, the IOM fills a unique role in employing the principle of recognition at the governmental level.

## Limitations of international organizations

There are many barriers that international organizations face in implementing planned relocation, highlighting the importance of partnership with other stakeholders. The limitations we delineate are not necessarily unique to international organizations but remain particularly challenging due to the scale at which they impact international organizations.

### Dependence on world order and global collaboration

The efficacy and power of international organizations are dependent on geopolitics and international relations broadly (Gabriela 2013). As such, their relative agency can be constrained just as much as it can be enabled, depending on the world order. Aside from funding restrictions, individual countries can choose whether or not to abide by agreements facilitated by international organizations due to a severe lack of enforcement mechanisms (Collingsworth 2002). This is especially concerning in cases of equitable planned relocation due to the trends in exclusionary ideology on the rise in the world. Planned relocation has the potential to magnify drivers of these politics (Smith 2007). Risks to equity lie in the potential for exclusion of affected parties from the decision-making process. Additionally, global coordination is dependent on the successful management of internal and external bureaucracies, which has the potential to stymie efforts (Bauer and Ege 2018).

### Conflicting priorities across stakeholders

International organizations take on many projects and priorities that have the potential to conflict with the long-term planning of relocation (Graham 2014). For instance, an organization might have an adaptation plan that implements agricultural technologies that will increase crop yields in the short term, whereas planned relocation to another area could increase crop yields in the long term. Governments, arguably the most important institutional partners in planned relocation, are likely to prioritize short-term benefits over long-term ones due to the political benefits of reducing short-term hardship (Boston 2014). In cases like this, the evaluation of multiple measures of well-being that takes into account differing time scales is likely to be overlooked.

### Lack of resources

Planned relocation is a resource-intensive process. Some international organizations can fund projects as intensive as this, such as the World Bank. Many other international organizations do not have the funding to do so, making them reliant on a patchwork of external funding sources, which could further introduce conflicting priorities (Kerlin 2013).

Without sufficient resources, applying the framework for equitable planned relocation becomes almost impossible. In order to recognize community members in the planning and decision-making process, many person-hours are needed to build and maintain relationships. Following this process, the creation of multiple options that take into account the various socio-cultural risk factors of the community members requires funding to organize and implement. Lastly, the longitudinal evaluation of multiple measures of well-being and the ability to act on those outcomes requires more person-hours and investment dollars than a typical project.

## Conclusion

As the hazards posed by climate change continue to rise in frequency and severity, planned relocation is an increasingly important adaptation mechanism. Especially in the current moment, the intersection of climate hazards, public health disasters, and border restrictions creates an urgent need for proactive action with a deliberate framing around equity in order to prevent the occurrence of compounded injustices. To this end, actors in the space should incorporate the framework for equitable planned relocation that we have proposed which encompasses comprehensive recognition of affected stakeholders in decision-making, consideration of socio-cultural risk factors relevant to relocation, and evaluation of multiple measures of well-being into their planned relocation efforts. International organizations have features that uniquely position them to implement the aforementioned principles. These include their abilities to agenda-set and fund, disseminate best practices and promote accountability, and facilitate coordination among relevant actors. It is critical to the success of equitable planned relocation efforts that international organizations do not act alone, but rather leverage their unique points of intervention in partnership with other governmental and non-governmental actors. Future areas of action include implementing, evaluating, and iterating upon the framework for equitable planned relocation.

## Abbreviations

CAT: Convention Against Torture and Other Cruel Inhuman or Degrading Treatment of Punishment
CEDAW: Convention on the Elimination of All Forms of Discrimination Against Women
CRC: Convention on the Rights of the Child
ICCPR: International Covenant on Civil and Political Rights
ICERD: International Convention on the Elimination of All Forms of Racial Discrimination
ICESCR: International Covenant on Economic, Social and Cultural Rights
ICMW: International Convention on the Protection of the Rights of All Migrant Workers and Members of Their Families
IDB: Interamerican Development Bank
IFRC: International Federation of Red Cross and Red Crescent Societies
INGO: International non-governmental organization
IO: International organization
IOM: International Organization for Migration
MDB: Multilateral development bank
NGO: Non-governmental organization
OECD: Organization for Economic Cooperation and Development
OHCHR: Office of the High Commissioner of Human Rights
PIDC: Pacific Inter-state Consultation Mechanism
UNFCCC: United Nations Framework Convention on Climate Change
UNHCR: United Nations High Commissioner for Refugees
UNICEF: United Nations Children’s Fund
USAID: United States Agency for International Development

## References

[CR1] Ajibade I (2019). Planned retreat in Global South megacities: disentangling policy, practice, and environmental justice. Climatic Change.

[CR2] Albert S, Bronen R, Tooler N, Leon J, Yee D, Ash J, Boseto D, Grinham A (2017). Heading for the hills: climate-driven community relocations in the Solomon Islands and Alaska provide insight for a 1.5 °C future. Regional Environmental Change.

[CR3] Almeida MV, Soares AL (2014). Knowledge sharing in project-based organizations: overcoming the informational limbo. International Journal of Information Management.

[CR4] Arnall A, Thomas DSG, Twyman C, Liverman D (2013). Flooding, resettlement, and change in livelihoods: evidence from rural Mozambique. Disasters.

[CR5] Baird IG, Shoemaker B (2007). Unsettling experiences: internal resettlement and international aid agencies in Laos. Development and Change.

[CR6] Barnett J (2016). A science of loss. Nature Climate Change.

[CR7] Bauer MW, Ege J (2018) The autonomy of international bureaucracies. In S. Kim, S. Ashley, & H. Lambright, Public administration in the context of global governance (63–84). Edward Elgar Publishing. 10.4337/9781783477807.00018

[CR8] Boh WF (2007). Mechanisms for sharing knowledge in project-based organizations. Information and Organization.

[CR9] Boston,J (2014) Governing for the future: how to bring the long-term into short-term political focus. Centre for Environmental Policy American University, 42

[CR10] Bowmer K (2007) Water and conflict resolution: from smoke filled rooms to public participation. Australian Stream Management Conference, Albury

[CR11] Bronen R (2011) Climate-induced community relocations: creating an adaptive governance framework based in human rights doctrine. NYU Review of Law & Social Change 35(357)

[CR12] Burns D, Heywood F, Taylor M, Wilde P, Wilson M (2004) Making community participation meaningful: a handbook for development and assessment. Policy Press

[CR13] Collingsworth T (2002). The key human rights challenge: developing enforcement mechanisms. Harvard Human Rights Journal.

[CR14] Dannenberg AL, Frumkin H, Hess JJ, Ebi KL (2019). Managed retreat as a strategy for climate change adaptation in small communities: public health implications. Climatic Change.

[CR15] Draper J, McKinnon C (2018). The ethics of climate-induced community displacement and resettlement. Wiley Interdisciplinary Reviews: Climate Change.

[CR16] Franta, B., Roa-Quiaoit, H. A., Lo, D., & Narisma, G. (2016). Climate disasters in the Philippines Harvard Kennedy School Belfer Center 27.

[CR17] Fraser N (2014) Justice interruptus: critical reflections on the “postsocialist” condition. Taylor & Francis https://books.google.com/books?id=ELZpAwAAQBAJ

[CR18] Gabriela, Sterian Maria. (2013). The role of international organizations in the global economic governance—an assessment. Romanian Economic and Business Review

[CR19] Georgetown Climate Center. (2020) Managed retreat toolkit

[CR20] Global Justice Center (2017) CEDAW casebank

[CR21] Graham ER (2014). International organizations as collective agents: fragmentation and the limits of principal control at the World Health Organization. European Journal of International Relations.

[CR22] Guaraldo Choguill M (1996). A ladder of community participation for underdeveloped countries. Habitat International.

[CR23] Gupta J (2016). Climate change governance: history, future, and triple-loop learning?: Climate change governance. Wiley Interdisciplinary Reviews: Climate Change.

[CR24] Hardoy J, Pandiella G, Barrero LSV (2011). Local disaster risk reduction in Latin American urban areas. Environment and Urbanization.

[CR25] Harris PG, Symons J (2010). Justice in adaptation to climate change: cosmopolitan implications for international institutions. Environmental Politics.

[CR26] Herrmann A, Sauerborn R (2018). General practitioners’ perceptions of heat health impacts on the elderly in the face of climate change—a qualitative study in Baden-Württemberg, Germany. International Journal of Environmental Research and Public Health.

[CR27] Hsiang SM, Sobel AH (2016). Potentially extreme population displacement and concentration in the tropics under non-extreme warming. Scientific Reports.

[CR28] IOM (2017) Migration, risk and resilience in the context of sudden and slow-onset disaster

[CR29] Kerlin JA (2013). Predicting variation in funding for international non-governmental organizations following three external events. Nonprofit Management and Leadership.

[CR30] Kuehn RR (2000). A taxonomy of environmental justice. Environmental Law Reporter.

[CR31] Kulp SA, Strauss BH (2019). New elevation data triple estimates of global vulnerability to sea-level rise and coastal flooding. Nature Communications.

[CR32] Leape, J. H. (2020). Winning the housing lottery in Rio de Janeiro: curse or cure? Massachusetts Institute of Technology.

[CR33] Malloy JT, Ashcraft CM (2020). A framework for implementing socially just climate adaptation. Climatic Change.

[CR34] McFarland KM (2017). Running in quicksand: environmental change, migration, and the policy imperatives. Development.

[CR35] McLeman R (2017). Thresholds in climate migration. Population and Environment.

[CR36] McLeman RA, Hunter LM (2010). Migration in the context of vulnerability and adaptation to climate change: insights from analogues: migration and adaptation to climate change. Wiley Interdisciplinary Reviews: Climate Change.

[CR37] McNamara KE, Bronen R, Fernando N, Klepp S (2018). The complex decision-making of climate-induced relocation: adaptation and loss and damage. Climate Policy.

[CR38] McWhirter R, Critchley C, Nicol D, Chalmers D, Whitton T, Otlowski M, Burgess M, Dickinson J (2014). Community engagement for big epidemiology: deliberative democracy as a tool. Journal of Personalized Medicine.

[CR39] Millner N, Peñagaricano I, Fernandez M, Snook LK (2019). The politics of participation: negotiating relationships through community forestry in the Maya Biosphere Reserve, Guatemala. World Development.

[CR40] Milton A, Rahman M, Hussain S, Jindal C, Choudhury S, Akter S, Ferdousi S, Mouly T, Hall J, Efird J (2017). Trapped in statelessness: Rohingya refugees in Bangladesh. International Journal of Environmental Research and Public Health.

[CR41] Mortreux C, Safra de Campos R, Adger WN, Ghosh T, Das S, Adams H, Hazra S (2018). Political economy of planned relocation: a model of action and inaction in government responses. Global Environmental Change.

[CR42] Moser C, Moser A (2010). Gender mainstreaming since Beijing: a review of success and limitations in international institutions. Gender & Development.

[CR43] Neal, William J., et al. (2017). Managed retreat. Encyclopedia of Engineering Geology, edited by Peter Bobrowsky and Brian Marker, Springer International Publishing, 1–7. 10.1007/978-3-319-48657-4_201-2.

[CR44] Niven RJ, Bardsley DK (2013). Planned retreat as a management response to coastal risk: a case study from the Fleurieu Peninsula, South Australia. Regional Environmental Change.

[CR45] Nygren A, Wayessa G (2018). At the intersections of multiple marginalisations: displacements and environmental justice in Mexico and Ethiopia. Environmental Sociology.

[CR46] Ocloo J, Matthews R (2016). From tokenism to empowerment: progressing patient and public involvement in healthcare improvement. BMJ Quality & Safety.

[CR47] OECD Development Assistance Committee (1992) Guidelines on aid and environment.

[CR48] OECD (2019) Responding to rising seas: OECD country approaches to tackling coastal risks, OECD Publishing, Paris, 10.1787/9789264312487-en

[CR49] OHCHR. (2020). The core international human rights instruments and their monitoring bodies.

[CR50] OPHI. (2015). Global multidimensional poverty index.

[CR51] Pacific Immigration Directors’ Conference (2010) Disaster response and the role of immigration.

[CR52] Paul, S. (1987). Community participation in development projects: the World Bank experience. The World Bank Group.

[CR53] Piggott-McKellar A, McNamara K, Nunn P, Sekinini S (2019). Moving people in a changing climate: lessons from two case studies in Fiji. Social Sciences.

[CR54] Pollack MA, Hafner-Burton E (2000). Mainstreaming gender in the European Union. Journal of European Public Policy.

[CR55] Rigaud, Kanta Kumari et al (2018) Groundswell: preparing for internal climate migration. World Bank, 19 Mar. 10.1596/29461.

[CR56] Robinson, W. C. (2003) Risks and rights: the causes, consequences, and challenges of development-induced displacement. The Brookings Institution.

[CR57] Schlosberg D (2012). Climate justice and capabilities: a framework for adaptation policy. Ethics & International Affairs.

[CR58] Schwerdtle P, Bowen K, McMichael C (2018). The health impacts of climate-related migration. BMC Medicine.

[CR59] Scott M, Lennon M, Tubridy D, Marchman P, Siders AR, Main KL, Herrmann V, Butler D, Frank K, Bosomworth K, Blanchi R, Johnson C (2020). Climate disruption and planning: resistance or retreat?. Planning Theory & Practice.

[CR60] Seck M (2018) World Bank board approves West Africa Coastal Areas (WACA) resilience investment project. The World Bank Group

[CR61] Smith PJ (2007). Climate change, mass migration and the military response. Orbis.

[CR62] The Interamerican Development Bank (1999) Involuntary Resettlement in IDB Projects

[CR63] The United Nations (2015) Sustainable development goals

[CR64] The World Bank (2004) Implementation completion report on a credit in the amount of SDR 1.5 million to the Lao People’s Democratic Republic for a district upland development and conservation project (No. 27881). World Bank Group

[CR65] The World Bank Group (1999) Lao PDR-district upland development and conservation project learning and innovation loan

[CR66] Thorpe A, Figge F (2018). Climate change and globalisation as ‘Double Exposure’: implications for policy development. Environmental Science & Policy.

[CR67] Tschakert P, Tutu R (2010) Solastalgia: environmentally induced distress and migration among Africa’s poor due to climate change. In: Afifi T, Jäger J (eds) Environment, Forced Migration and Social Vulnerability 57–69. Springer. 10.1007/978-3-642-12416-7_5

[CR68] Tuhkanen H, Boyland M, Han G, Patel A, Johnson K, Rosemarin A, Lim Mangada L (2018). A typology framework for tradeoffs in development and disaster risk reduction: a case study of Typhoon Haiyan recovery in Tacloban, Philippines. Sustainability.

[CR69] UNHCR (2011) UNHCR resettlement handbook. Division of International Protection

[CR70] UNHCR Brookings Institution, & Georgetown University (2016) A toolbox: planning relocations to protect people from disasters and environmental change

[CR71] United Nations (2017) UN Climate Change Annual Report 2017. United Nations Framework Convention on Climate Change. https://unfccc.int/resource/annualreport/

[CR72] United States Government Accountability Office (2020) A climate migration pilot program could enhance the nation’s resilience and reduce federal fiscal exposure (No. 20–488)

[CR73] Vithanagama, R., Mohideen, A., Jayatilaka, D., & Lakshman, R. (2015) Planned relocations in the context of natural disasters: the case of Sri Lanka. 37

[CR74] Walls ML, Whitbeck LB (2012). The intergenerational effects of relocation policies on indigenous families. Journal of Family Issues.

[CR75] Warner, K., Afifi, T., Kälin, W., Leckie, S., Ferris, B., Martin, S. F., & Wrathall, D. (2013). Changing climate, moving people: framing migration, displacement and planned relocation. UN University

[CR76] Wang S,He J (2019) UNICEF’s global social protection programme framework. UNICEF.

[CR77] Young, Iris (1990) Justice and the politics of difference. Princeton University Press

[CR78] World Bank Group (2019) The World Bank Group’s action plan on climate change adaptation and resilience. World Bank. http://documents1.worldbank.org/curated/en/519821547481031999/The-World-Bank-Groups-Action-Plan-on-Climate-Change-Adaptation-and-Resilience-Managing-Risks-for-a-More-Resilient-Future.pdf

[CR79] Zakus JD, Lysack CL (1998). Revisiting community participation. Health Policy and Planning.

[CR80] Zhang Y, Fung T (2013). A model of conflict resolution in public participation GIS for land-use planning. Environment and Planning B: Planning and Design.

